# Transition-metal-nitride-based thin films as novel energy harvesting materials

**DOI:** 10.1039/c5tc03891j

**Published:** 2016-02-19

**Authors:** Per Eklund, Sit Kerdsongpanya, Björn Alling

**Affiliations:** a Thin Film Physics Division , Linköping University , IFM , 581 83 Linköping , Sweden . Email: perek@ifm.liu.se; b Max-Planck-Institut für Eisenforschung GmbH , D-40237 Düsseldorf , Germany

## Abstract

The last few years have seen a rise in the interest in early transition-metal and rare-earth nitrides, primarily based on ScN and CrN, for energy harvesting by thermoelectricity and piezoelectricity. This is because of a number of important advances, among those the discoveries of exceptionally high piezoelectric coupling coefficient in (Sc,Al)N alloys and of high thermoelectric power factors of ScN-based and CrN-based thin films. These materials also constitute well-defined model systems for investigating thermodynamics of mixing for alloying and nanostructural design for optimization of phase stability and band structure. These features have implications for and can be used for tailoring of thermoelectric and piezoelectric properties. In this highlight article, we review the ScN- and CrN-based transition-metal nitrides for thermoelectrics, and drawing parallels with piezoelectricity. We further discuss these materials as a models systems for general strategies for tailoring of thermoelectric properties by integrated theoretical–experimental approaches.

## Abstract 2

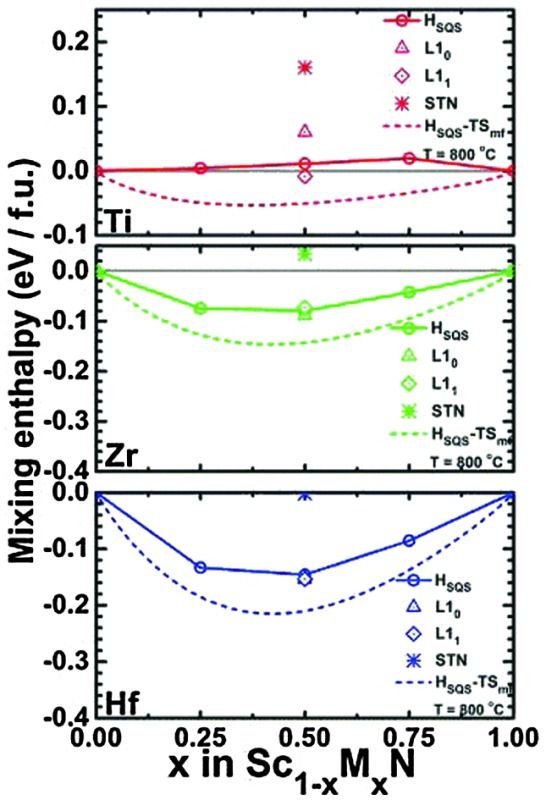
We review experimental and theoretical research on ScN- and CrN-based transition-metal nitride materials for thermoelectrics, drawing parallels with piezoelectricity.

## I. Introduction

In the last few years, the early transition-metal and rare-earth nitrides, primarily based on ScN and CrN, have emerged as an unexpected class of materials for energy harvesting by thermoelectricity and piezoelectricity and more generally for conversion of heat or mechanical energy to electricity. Largely ignored for these purposes until around 2010, this class of materials is now on the rise because of a number of fundamental advances, among those the discoveries of exceptionally high piezoelectric coupling coefficient in (Sc,Al)N alloys^1^ and of remarkably high thermoelectric power factors of ScN-based^2,3^ and CrN-based^4,5^ thin films. These materials also constitute well-defined model systems for investigating thermodynamics of mixing for alloying and nanostructural design for optimization of phase stability and band structure. These features have implications for and can be used for tailoring of thermoelectric and piezoelectric properties.

The process of energy harvesting is the capture of energy from ambient sources and storage and/or application for use as power sources. There is a wide range of ambient sources, including solar, wind, electromagnetic radiation, mechanical (kinetic) energy, and thermal energy. Energy harvesting differs conceptually from, *e.g.*, oil and coal power, fuel cells, or batteries, that involve active combustion of a fuel or conversion of stored chemical energy to electricity. Furthermore, the term energy harvesting is typically reserved for capturing energy for powering small, low-power devices, usually off-grid or otherwise autonomous. The term does not include, *e.g.*, solar-power and wind-power plants, although the fundamental concept is the same.

In this highlight article, we review the ScN- and CrN-based transition-metal nitrides for energy-harvesting applications by thermoelectrics (harvesting of ambient heat), drawing parallels with piezoelectricity (harvesting of mechanical vibrations). It is further intended as an example of general strategies for tailoring of thermoelectric properties by integrated theoretical-experimental approaches.

## II. Brief introduction to thermoelectricity

Thermoelectric devices harvest thermal energy (temperature gradients) into electricity, and can also be used for environmentally friendly refrigeration, without moving parts or malign liquids or gases. Most other conversion systems (such as power plants) become less efficient as they are scaled down in size and power, but thermoelectrics benefit from low- to medium-power and -size application. Thus, potential contributions of thermoelectrics are in applications with relatively low power levels used in large numbers (for example in personal computers, automotive applications, and consumer electronics). The efficiency of a thermoelectric material at a temperature *T* is related to the dimensionless figure of merit *ZT*, where *Z* = *S* ^2^ *σ*/*κ*. Here, *σ* and κ are the electrical and thermal conductivities, respectively, and *S* is the Seebeck coefficient Δ*V*/Δ*T*, *i.e.*, the voltage in response to a temperature gradient. The thermal conductivity is given by *κ = κ*_l_ + *κ*_e_, where the subscripts l and e denote the lattice (phonon) and electron contributions, respectively. Thus, a high *ZT* requires a good electrical conductor with high Seebeck coefficient but low thermal conductivity.^6^ For traditional thermoelectric materials like tellurides and antimonides, *ZT* ≈ 1 at room temperature. At first glance, it seems easy to increase *ZT* by, *e.g.*, increasing the conductivity by a factor of 2–4. However, basic transport theory in solids implies that *S*, *σ*, and *κ* are interrelated; increasing *σ* by increasing the charge carrier concentration results in lower *S* and increased *κ*, yielding no improvement in *ZT*.

The delicate interdependence of the three parameters *S*, *σ*, and *κ* requires novel approaches to advance the field of thermoelectrics, which has led to extensive efforts on nanostructural design.^7,8^ Predictions in the mid-1990s suggested that *ZT* could be enhanced by quantum confinement.^9^ In 2001, thermoelectric superlattice devices of Bi_2_Te_3_/Sb_2_Te_3_ with remarkably high *ZT* (∼2.4) caused a surge of interest.^10^ Materials with such high *ZT*, however, are restricted to laboratory devices, which has prevented their success in practice.^11^ At present, the main achievement of nanostructuring is reduction of the lattice thermal conductivity^12,13^ rather than improvements due to quantum confinement of charge carriers.^14,15^

There is therefore a need to introduce mechanisms that, in addition to reducing *κ*, also enhance the thermoelectric power factor (*S* ^2^ *σ*). One innovative approach is to consider theoretically what band structure a hypothetical material should have to maximize *ZT*. Mahan and Sofo^16^ predicted this in the 1990s, and others have more recently refined the picture.^17^ For a given *κ*_l_, the ideal transport-distribution function that maximizes *ZT* is a bounded delta function, approximately realized in practice as a sharp function with a large slope in the density of states (DOS) at the Fermi level *E*_F_.^9,15^ These approaches are the base for modern strategies for the development of thermoelectrics: band-structure optimization to emulate the ideal band structure, combined with nanostructural design to reduce the thermal conductivity.

## III. The early transition-metal nitrides

### A. Overall trends

The early transition-metal nitrides – and their alloys – based on group-4 (Ti, Zr, and Hf) or group-5 (V, Nb, and Ta) metals, are long-established in applications as hard, wear-resistant coatings, with TiN being the archetype. While they are hard and exhibit other typical ceramic properties, these nitrides are metallic in nature with respect to electrical properties and in fact very good conductors, with typical resistivities in the approximate range 10–30 μΩcm (in comparison, noble metals have resistivities of a few μΩcm, and Ti metal about 40 μΩcm).^18^ Thus, they find extensive use as conducting permanent contact layers and diffusion barriers in microelectronics.

Reducing the valency by one from Ti or increasing it by one from V, *i.e.*, moving to groups 3 or 6 in the periodic table, results in drastically altered properties. The cubic rock-salt-structured ScN and CrN are both narrow-bandgap semiconductors. Sc has three valence electrons that together with the three 2p valence electrons of N complete the filling of the bonding states formed by nearest neighbor hybridization of mainly N 2p and Sc 3d e_g_ with some Sc 4s character. The 3d t_2g_-orbitals, on the other hand, are completely empty, in contrast to the group-4 and -5 transition metals, and the Fermi level drops below the conduction band edge, causing ScN to become semiconducting. In CrN, with three more electrons than ScN, the non-bonding Cr 3d t_2g_-band is half-filled. This causes a spin splitting of the band which give Cr atoms of CrN a distinct local magnetic moment approaching 3 *μ*_B_.^19^ As a consequence, also in this material, the Fermi level falls into a bandgap, this time between occupied spin-up non-bonding Cr 3d t_2g_ and an unoccupied mixture of mostly anti-bonding spin-up Cr 3d e_g_, and non-bonding spin-down Cr 3d t_2g_.^20,21^

**Fig. 1:**
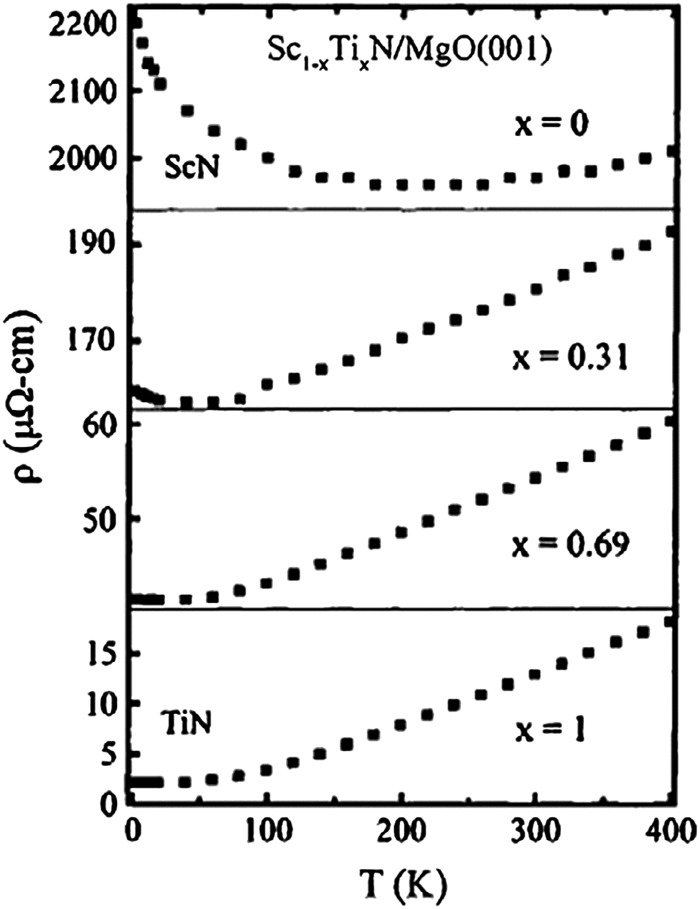
Electrical properties of ScN, TiN, and ScTiN alloys. From Gall *et al.* ^22^ (Copyright American Institute of Physics, used with permission).

The effect on resistivity is illustrated in Fig. 1, from an early study by Gall *et al.* ^22^ who investigated Ti_*x*_Sc_1–*x*_N epitaxial thin films. Pure TiN is a good conductor and exhibits a typical room-temperature resistivity value around 20 μΩcm, and the archetypical metallic temperature dependence with constant resistivity at low temperature dominated by scattering from vacancies, defects, and impurities. As temperature increases, the resistivity increases linearly with the scattering dominated by electron–phonon coupling. For low Sc content in the Ti_*x*_Sc_1–*x*_N alloy, this behavior is initially retained, but for higher Sc content, an increase in resistivity is observed at cryogenic temperature and for pure ScN this effect is dominant indicating semiconducting behavior. It needs to be stressed, though, that this is highly dependent on impurities and dopants.

### B. ScN

ScN, like the group-4 and group-5 transition-metal nitrides, is an interstitial (cubic NaCl structure) nitride following Hägg's rule; that interstitial nitrides and carbides are formed when the radii ratio between the nonmetal and metal atoms is smaller than 0.59.^23^ ScN has similar hardness as the other transition-metal nitrides, 21 GPa, and is stable at high temperature (melting point 2550 °C), while it is prone to oxidation if used in air above 600 °C.^24–26^

As stated above, ScN is a narrow-bandgap semiconductor. The fundamental bandgap is ∼0.9 eV and the direct (optical bandgap) is ∼2.1 eV. This was, however, a topic of debate for a rather long time with numerous studies yielding conflicting results as to whether ScN was a semiconductor, a semimetal or even a metal.^27–30^ The reason for these discrepancies are that it is challenging to produce pure ScN. Sc has a high affinity to oxygen^31^ and, if not synthesized in a pure ultrahigh-vacuum environment, can readily contain large amounts of oxygen impurities, as well as contaminations from residual hydrocarbons. Free carriers from impurities can result in large inaccuracies in determinations of optical bandgaps.^30^ Furthermore, processing of scandium ore involves a purification step with fluoride reduction,^32^ which results in scandium raw materials often containing fluorine impurities.

There are relatively many studies on thin-film growth of ScN. Among the methods used, magnetron sputter deposition,^2,22,30,33–35^ chemical vapor deposition^24,36^ and molecular beam epitaxy^37–42^ are the most common. Irrespective of method, the aspects of reactivity and oxygen and/or fluorine uptake (or other impurities) are essential in thin-film growth of ScN, stressing the need for a pure environment.

**Fig. 2:**
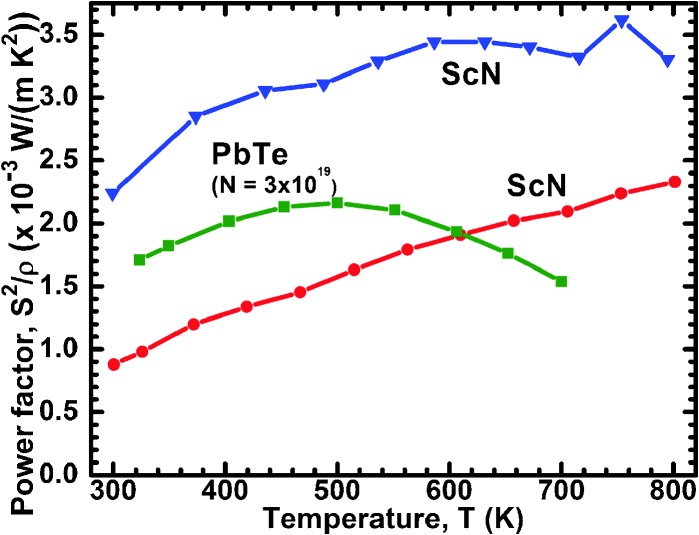
Thermoelectric power factor *S* ^*2*^ *σ* of ScN (the bottom curve shows our first data from ref. 2 and the top curve are the data of Burmistrova *et al.*).^3^ The example data for PbTe are adapted from Sootsman *et al.* ^43^

For a transition-metal nitride, ScN exhibit an anomalously high thermoelectric power factor^2,3^ *S* ^2^ *σ. S* ^2^ *σ* is in the range 2.5–3.3 W m^–1^ K^–2^, well on par with established thermoelectric materials such as PbTe.^43^ This is illustrated in Fig. 2, where ScN (our data from ref. 2 and the results of Burmistrova *et al.* ^3^) are shown in relation to a typical value for n-type PbTe. In comparison, the power factor of Bi_2_Te_3_ is somewhat higher at above 4 W m^–1^ K^–2^. The thermal conductivity of ScN, though, is much higher than for these tellurides, in the range 8–12 W m^–1^ K^–1^,^3,24,44^ and would need to be drastically reduced to enable application of ScN as a thermoelectric material; strategies for addressing this are discussed in Section V below. These tellurides are benchmark thermoelectric materials. Nonetheless, the scarcity^45^ of Te as well as legislative restrictions on the use of Pb limits their applicability outside niche applications. Hence, much effort is devoted to developing alternative materials. The early transition-metal nitrides are a class that was not much considered for this purpose until just a few years ago. From an application point-of-view, CrN-based materials are closer to application than ScN-based ones, since the former are abundant, relatively inexpensive and can readily be made in large quantities by standard processing techniques both in thin films and bulk.

**Fig. 3:**
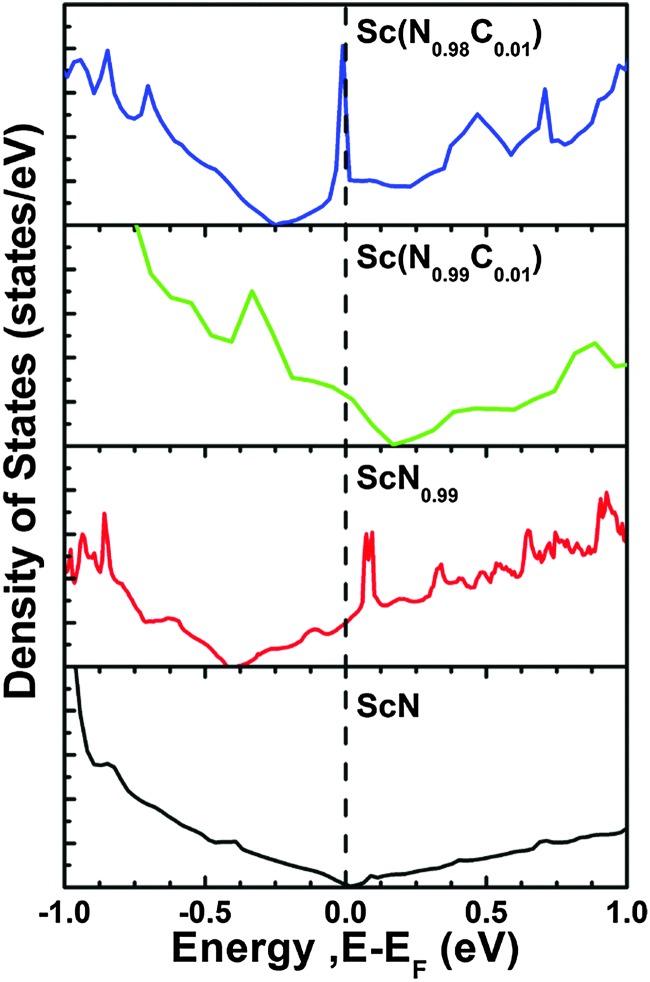
Example of effects of vacancies and dopants on the band structure of ScN (adapted from ref. 46). Bottom: Pure, stoichiometric ScN (note the inaccurate bandgap determination with GGA). Second from bottom: 1% N vacancies. Second from top: 1% C dopants. Top: Both C dopants and N vacancies.

The unexpectedly high thermoelectric power factor of ScN can be explained based on band-structure features caused by impurities. A conceptual illustrative example is shown in Fig. 3 (adapted from ref. 46), where first-principles calculations show that the combination of (in this example) C dopants and N vacancies in ScN introduces a sharp variation in the density of states at the Fermi level. As described in Section II, to maximize *ZT*, the transport-distribution function should be a bounded delta function (for a given phonon *κ*), realized in practice as a large slope in the density of states near the Fermi level. Thus, the electronic structure of ScN – including vacancies and impurities n the level of ∼1 at% – can mimic the ideal theoretical transport-distribution function, yielding a high power factor.^46,47^ The same conclusions are drawn from calculations with O or F dopants.^3,46^

### C. CrN

CrN is well known from applications as hard coatings, given its high hardness of 28 GPa and good resistance to wear and corrosion.^48–58^ It can also be readily synthesized in bulk.^59,60^ In addition to these properties, CrN exhibits a magnetic phase transition.^19,61,62^ Above the Néel temperature (*T*_N_) of 286 K, CrN is paramagnetic with cubic (NaCl) crystal structure with a lattice parameter reported to range from 4.135 to 4.185 Å.^63–66^ Below this temperature, the structure is antiferromagnetic and orthorhombic.

The electrical properties of CrN can vary greatly; for example, various studies show resistivity values ranging from 1.7 to 350 mΩcm.^4,55,60,67^ As for the reported temperature-dependent behavior of the electrical resistivity around the cubic-to-orthorhombic phase transition,^68^ there is typically a jump in resistivity between these two semiconducting phases, though there are reports of metallic behavior for the orthorhombic phase.^69^ Part of this apparent discrepancy can be attributed to CrN being a narrow-bandgap semiconductor, where the presence of N vacancies may act as effective dopants yielding high electron concentrations and metallic-like behavior below the Néel temperature.^70^ In epitaxial thin films, it is possible to stabilize the cubic phase and suppress the phase transition to the orthorhombic phase^71–73^ In addition, Gall *et al.* ^72^ suggested that the conducting behavior in CrN films is a hopping conduction mechanism, and the band gap of CrN depends on the correlation energy.

For thermoelectric properties, CrN exhibits high Seebeck coefficients of typically around 135 μV K^–1^ around room temperature and up to 200 μV K^–1^ at 600 K.^60^ Also, its thermal conductivity is moderate at ∼1.7 W m^–1^ K^–1^ (∼1/5 of that of ScN). Nonetheless, the electrical resistivity is relatively high; in pure form, because the localized 3d orbitals of Cr give large effective masses causing high Seebeck coefficients and resistivities. This was recently addressed by Quintela *et al.* ^5^ who annealed as-deposited films in ammonia gas for 2 h at 800 °C to ensure that the films were fully stoichiometric and to improve the crystalline quality; yielding a large improvement in Seebeck coefficient and a hundredfold reduction in resistivity.

This further underscores that the early transition-metal nitrides hold unexpected promise as novel thermoelectric materials. However, in their pure form, neither ScN nor CrN are likely to reach all the way; experimental strategies and theoretically guided design approaches for reduction of the thermal conductivity with retained or increased power factors are needed.

## IV. Theoretical methodology

Electronic structure calculations based on density functional theory (DFT)^74,75^ and standard approximations for the exchange–correlation energies like the local density approximation (LDA) and the different flavors of the generalized gradient approximation (GGA)^76^ are very efficient and most often accurate tools for phase stability and ground state related properties for most classes of materials. However, both the existing cases where this framework is insufficient in treatment of the quantum electronic problem, and perhaps more frequently, the neglect in theoretical studies of relevant vibrational, magnetic, and structural disorder, present in reality, is an obstacle in first-principles based calculations for real-world materials science problems. In fact, the early 3d transition metal nitrides constitute an interesting illustration of several of these challenges. In ScN, calculations of the bandgap using the Kohn–Sham orbital gap in GGA gives basically a zero-gap semiconductor, an underestimation effect well known and found for practically all semi-conductors. This is seen in the bottom curve in Fig. 2, from ref. 46. In VN, the rock-salt structure observed at room temperature and above is actually dynamically stabilized by anharmonic lattice vibrations and unstable at low temperature.^77^ This can lead to unphysical large atomic relaxation effects in VN-based systems if static, 0 K, calculations are performed without symmetry constraints, *e.g.*, with point defects or alloys. Finally, in CrN, the effect of strong electron correlation is treatable on the level of LDA+U.^70,78,79^ However, most importantly the magnetic degree of freedom has caused considerable debate. In particular, the bulk modulus of paramagnetic rock-salt phase of CrN has been modeled using non-spin polarized calculations.^80^ However, the existence of local finite Cr moments, also above the magnetic ordering temperature, is crucial to consider in the theoretical modeling,^81^ otherwise the bulk modulus is greatly overestimated. This model, including local Cr moments in the paramagnetic phase, were later independently confirmed by new sets of experiments.^82^ The simultaneous presence of lattice vibrations and disordered magnetic moments in paramagnetic rock salt CrN causes further challenges for quantitative modeling. In fact, due to this complexity, the material has become a benchmark case for theoretical method development in the field.^83–86^ A similar finding of the importance of magnetism was also made for the bulk modulus of Cr_2_AlC and other Cr-containing so-called MAX phases.^87^ The crucial importance of the details of the nitrogen content and oxygen contamination levels pointed out in the previous section, also causes concern for the theoretical modeling of CrN as it couples with the vibrational and magnetic degrees of freedom.^88^

The current outstanding issue in this line of theoretical development, is associated with the unclear, material-specific, timescale for the propagation of the magnetic state as compared to the dynamics of the lattice. Recently, several methodological obstacles have been overcome, and methods for constrained local moments calculations^89^ and the derivation of Heisenberg-type exchange interactions,^90^ within the supercell based plane-wave electronic structure frameworks needed for *ab initio* molecular dynamics now exist. The stage is set for a direct combinations of molecular and spin dynamics in an effective *ab initio* manner with minimum or no free parameters. It would open up for first principles based calculations of lattice and magnetic thermal conductivity in the paramagnetic phase of magnetic materials, such as CrN.

In the case of first-principles modeling of substitutionally disordered nitride alloys, *e.g.*, Cr_1–*x*_Al_*x*_N and Ti_1–*x*_Al_*x*_N,^91^ the configurational problem arise as the crystallographic unit cell is no longer sufficient to describe the material. In a completely random alloy, the components are stochastically distributed on the lattice sites, metal sublattice in the case of a nitride alloy, implying lack of long range order and existence of many different local chemical environments of the atoms. The most reliable method to model such materials is the special quasirandom structure (SQS) method^92^ introduced for transition metal nitride alloys in a study of Ti_1–*x*_Al_*x*_N.^93^ Using the SQS approach the mixing thermodynamics of the alloys can be directly modeled within a mean-field approximation for the configurational entropy. Also, *e.g.*, the piezoelectric properties can be calculated directly.^94,95^ For the alloys of transition metal nitrides, and group-13 nitrides like AlN, such modeling has revealed important information about the mixing trends, *i.e.*, if the supersaturated alloys obtained in the out-of-equilibrium synthesis at low temperature, will phase separate, order, or stay as a solid solutions when subject to the temperatures needed to induce metal-sublattice diffusion, *e.g.*, in several (Sc,M)N^96^ and (Cr,M)N^97^ alloys.

It should be noted that real alloys under equilibrium conditions always display some degree of partial short-range ordering or, short-range clustering. This can of course also be the case for a metastable supersaturated solid solution grown with out-of-equilibrium techniques. However, in lack of *a priori* knowledge of such tendencies, the ideal random SQS approach is a well-defined, unbiased starting point for *e.g.* more intricate cluster-expansion approaches of the configurational thermodynamics.^98^ Outstanding issues here include the difficulty to include vibrational free energy, and in particular anharmonic contributions, into the configurational thermodynamics analysis in an accurate and efficient manner.^99^

With a reliable state-of-the art theoretical description of the materials equilibrium properties, the door opens for accurate calculations of properties. However, such calculations involves drastically different levels of complexity depending on which property that is needed. The properties needed for predicting the piezoelectric response of a material are second-order strain-derivatives of ground state energies, elastic constants, and first-order strain-derivatives of polarization.^100^ These are relatively straightforward to calculate accurately from first-principles with the complexities arising mostly from their tensorial nature, where care must be taken in the case of disordered alloys.^101^

The properties needed for understanding thermoelectric behavior of a material, on the other hand, are quite challenging to derive directly and accurately from first-principles, because the thermoelectric figure of merit includes both electronic and thermal transport and the entropy involves non-equilibrium transport processes. *Ab initio* calculation of thermoelectric parameters is addressed by Boltzmann transport theory,^16,102^ but involves an unknown scattering parameter, the relaxation time *τ*. For the Seebeck coefficient (and Hall coefficient), *τ* cancels out if it is isotropic and constant with respect to energy. However, electrical and (electronic) thermal conductivities can only be determined either as a function of *τ* or by fitting to experimentally determined values^103^ of *τ*, placing a substantial limitation on these computational approaches. Such calculations of the latter parameters are therefore not truly *ab initio* but restricted to materials for which experimental data of *τ* (or parameters from which *τ* can be calculated) are available. Ongoing method development is therefore devoted to finding methods for computing these from first principles. Examples are the recent work of Faghaninia *et al.* ^104^ who derived an *ab initio* approach for computing these properties in the low-electric-field limit, and efforts to incorporate low-temperature effect of phonon drag.^105,106^

## V. Ternary systems

As proposed above, ScN and CrN are promising for thermoelectrics and based on cheap raw materials, while a reduced thermal conductivity of ScN or reduced electrical resistivity of CrN would be required for actual applications. This can be addressed in a Cr_1–*x*_Sc_*x*_N solid solution, which is thermodynamically stable at high temperature in cubic NaCl-structured form.^107^ The fact that the 3d orbitals in Sc are empty can be exploited as a means of delocalizing the electrons in 3d orbitals, resulting in electrical conductivity reduction^107^ and possibly also thermal-conductivity reduction due to alloy scattering. We have recently shown that the Seebeck coefficient of Sc-rich Cr_1–*x*_Sc_*x*_N solid solution epitaxial thin films does indeed increase compared to pure ScN and that the thermoelectric properties of CrN are largely retained in Cr-rich Cr_1–*x*_Sc_*x*_N solid solutions.^107^

ScN-based solid solutions are further important, because of the interest caused by the exceptionally high piezoelectric coupling coefficient in (Sc,Al)N alloys.^1,108^ (Sc,Al)N and (Sc,Ga)N alloys were recently reviewed by Moram and Zhang^109^ and the reader is referred there. (Sc,Mn)N was investigated by Saha *et al.* ^110^

Alloy scattering is one of the standard strategies for thermoelectric materials for reduction of the lattice thermal conductivity; other approaches are superlattices, nanoinclusions, or grain boundaries.^6,111–114^ Furthermore, the peaks in the density of states at the Fermi level causing high Seebeck coefficient is traditionally associated with reduced thermodynamic stability.^115,116^ For this reason, the search for optimal thermoelectric materials may be fruitful among metastable materials synthesized with far-from-equilibrium techniques, such as magnetron-sputtered metastable nitride thin film alloys, with the reservation that the metastable nature of such materials would place a limit on high-temperature long-term use.

ScN- and CrN-based systems are interesting model systems for these general research questions.^96^ In particular, Sc is naturally isotope-pure, thus lacking isotope reduction of thermal conductivity. Consequently, the possibilities to substantially reduce the thermal conductivity by alloying or nanostructural engineering are particularly promising in this material. If the thermal conductivity can be reduced, ScN-based materials could potentially be applied at elevated temperatures, where bulk diffusion can be activated and the thermodynamics of mixing between ScN and the alloying or superlattice component becomes relevant. Superlattices might intermix, alloys could order or phase-separate, and nanostructures might be dissolved in the matrix. All these processes will most likely affect thermoelectric properties.

**Fig. 4:**
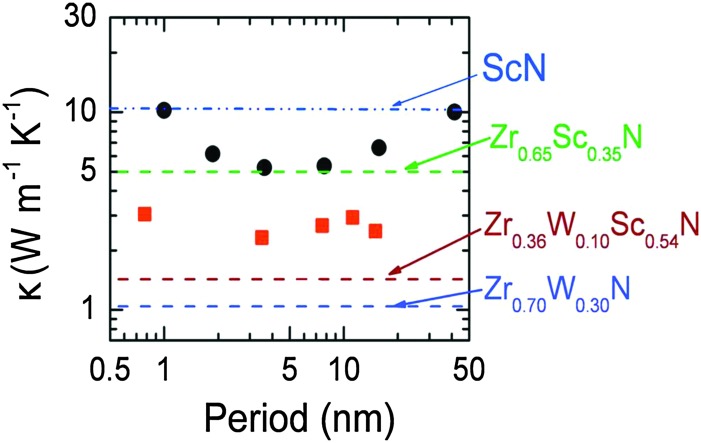
Illustration of superlattice reduction in thermal conductivity, from Rawat *et al.* ^44^ Cross-plane thermal conductivity of 300 nm thick ZrN/ScN (dots) and Zr_0.64_W_0.36_N/ScN (squares) multilayers. Superimposed on the plot are horizontal lines corresponding to the experimentally determined lattice component of thermal conductivity, *i.e.*, the alloy limit of different alloys of ZrN, ScN, and W_2_N. (Copyright American Institute of Physics, used with permission).

Superlattices are of great interest for thermoelectrics, since they may allow for both the reduction of the lattice thermal conductivity and the quantum confinement of electrons. The first thermoelectric superlattice devices were made from combinations of the semiconductors Bi_2_Te_3_/Sb_2_Te_3_.^117^ A different approach is to combine the high electron concentrations of ultrathin metallic layers (*e.g.*, TiN or ZrN) inserted between semiconductor barriers (*e.g.*, CrN, ScN), the sharp asymmetry in the conduction electron distribution near the Fermi energy may be achieved for possible substantial improvements in *ZT*.^118^ Furthermore, it has been demonstrated that the high interface density in a superlattice can reduce the thermal conductivity in ScN/(Zr,W)N superlattices.^44^ This is illustrated in Fig. 4, from Rawat *et al.* ^44^

**Fig. 5:**
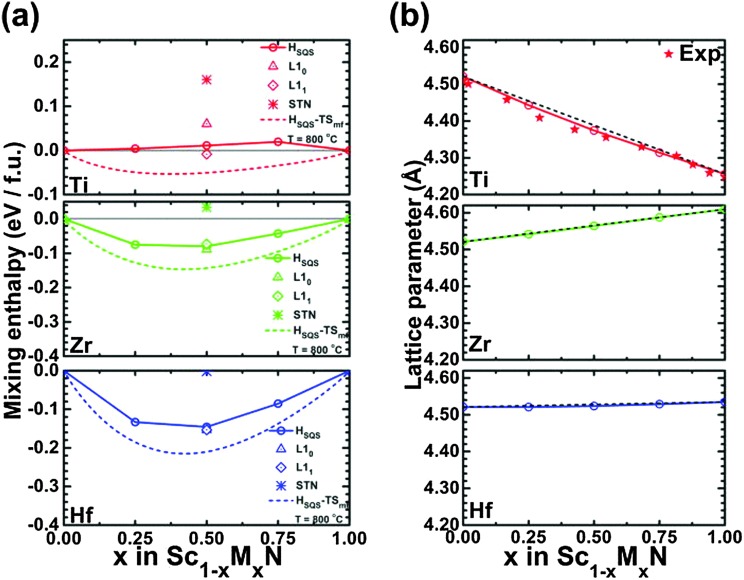
(a) Comparison of the calculated mixing enthalpies of substitutionally disordered solid solution, ordered solid solutions and ScTaN_2_-type structure phase of Sc_1–*x*_M_*x*_N, as a function of MN content where M = Ti, Zr, and Hf, respectively. (b) Calculated equilibrium lattice parameter for rocksalt (B1) Sc_1–*x*_M_*x*_N solid solution as a function of MN content where M = Ti, Zr, and Hf, respectively. The black line indicates Vegard's rule. For (Sc,Ti)N, experimental data from Gall *et al.* ^22^ are shown with stars. From ref. 96 (Copyright American Institute of Physics, used with permission).

A theoretically guided approach to implementing these strategies are to use density functional theory calculations to investigate the effect of mixing thermodynamics in order to determine phase stability of ScN-based solid solutions of relevance for lattice thermal conductivity reduction. Our results demonstrated^96^ that at 800 °C the free energy of mixing for (Sc,Y)N, (Sc,La)N, (Sc,Gd)N, and (Sc,In)N exhibits a thermodynamic tendency for phase separation at high temperature. In addition, for the 50 : 50 Sc : M (M = V, Nb, or Ta) ratio, the (Sc,V)N, (Sc,Nb)N, and (Sc,Ta)N exhibit a stable ternary inherently nanolaminated phase^119^ with the ScTaN_2_-type structure. On the other hand, at 800 °C, the (Sc,Ti)N, (Sc,Zr)N, (Sc,Hf)N, and (Sc,Lu)N are thermodynamically stable in disordered B1 (NaCl) solid solutions, rather than in the ordered solid solutions which are stable at 0 K. This last point is shown in Fig. 5 (from ref. 96), which shows (Fig. 5(a)) a comparison of the calculated mixing enthalpies of substitutionally disordered solid solution, ordered solid solutions and ScTaN_2_-type structure phase of (Sc,M)N, as a function of MN content where M = Ti, Zr, and Hf, and (Fig. 5(b)) calculated equilibrium lattice parameter for the rocksalt (B1) solid solution as a function of MN content.

These results enabled us to suggest suitable materials for the different possible strategies for reduction of the lattice thermal conductivity of ScN. Since the heavy element Lu has a mixing tendency with ScN and has the same number of valence electrons, it is an appropriate choice for solid-solution reduction of the thermal conductivity. YN, LaN, GdN, AlN, GaN, InN, have a thermodynamic tendency for phase separation with ScN and thus constitute good alloying elements if decomposition to form nanoinclusions is the strategy. The three former can be used for superlattices, since they in addition are isostructural with ScN. That, in combination with the thermodynamic tendency for phase separation, would render the superlattice structure stable. The wurtzite AlN, GaN, and InN are not suited for this purpose because of the difference in crystal structure, but their alloys with ScN or other NaCl-structure nitrides can be, as described below.^120–122^ The mixing thermodynamics of these alloy systems can be understood by considering the effect of the factors of volume mismatch, favoring phase separation, and an electronic structure effect of delocalization of extra d-electrons to empty Sc 3d-t_2g_ states, favoring mixing.

Important experimental demonstrations of these principles are the works of Saha *et al.* who stabilized cubic (Sc,Al)N lattice-matched^121^ to TiN and (Ti,W)N in TiN/(Sc,Al)N and (Ti,W)N/(Sc,Al)N superlattices exhibiting enhanced hardness^122^ and large reduction in thermal conductivity.^123^ Here, the combination of superlattice scattering and heavy-element alloying (with W) allowed for thermal conductivities down to 1.7 W m^–1^ K^–1^ (compared to 8–10 W m^–1^ K^–1^ for pure ScN). Nonetheless, as discussed above, these superlattice structures are metastable and limited in use to the medium-temperature range. Around 800 °C, where bulk diffusion becomes dominant, their long-term thermal stability would be compromised due to intermixing. This was demonstrated by Schreoder *et al.* who showed that TiN/(Sc,Al)N superlattices intermix heavily at elevated temperature.^120^

Finally, we note that recently theoretical calculations has been used to suggest further alternatives to the ScN and CrN based semiconducting systems. In particular, by combining group-4 transition metals with a group-2 alkaline-earth metal in equal amounts, novel semiconducting systems have been predicted, like Ti_0.5_Mg_0.5_N^124^ and the wurtzite-structure (TM_0.5_,M_0.5_)_*x*_Al_1–*x*_N alloys are investigated for piezoelectric properties.^125,126^ These recent studies demonstrate the superior speed with which computationally based approaches can scan large pools of complex, uncharted materials and suggest candidates for a given property, before experimental verification is pursued. This emphasizes the promise for a future of theoretically driven materials discoveries, at least in the cases where the theoretical accuracy and methodological reliability is well established.

## VI. Concluding remarks

We have reviewed the present state of research on early transition-metal nitrides, primarily based on ScN and CrN, for thermoelectric energy harvesting. These materials also constitute well-defined model systems for theory-guided approaches investigating thermodynamics of mixing for alloying and nanostructural design for optimization of phase stability and band structure, in order to improve thermoelectric properties. This is most notable as thermoelectric properties *per se* – unlike piezoelectric properties – are challenging to reliably calculate by *ab initio* methods. This can be used to guide the implementation of strategies for reduction of the lattice thermal conductivity; alloy scattering, superlattices, and nanoinclusions.
